# Investigation of cortical cholinergic projection integrity in relation to longitudinal cognitive performance in Parkinson's disease

**DOI:** 10.1002/alz70856_105399

**Published:** 2026-01-09

**Authors:** Nicola M Slater, Daniel J Myall, Kyla‐Louise Horne, Campbell J Le Heron, Ross J Keenan, Ian J Kirk, Wassilios G Meissner, Tim J Anderson, Tracy R Melzer, John C Dalrymple‐Alford

**Affiliations:** ^1^ University of Otago, Christchurch, New Zealand; ^2^ New Zealand Brain Research Institute, Christchurch, New Zealand; ^3^ Christchurch Hospital, Christchurch, Canterbury, New Zealand; ^4^ University of Canterbury, Christchurch, New Zealand; ^5^ Radiology Holding Company New Zealand, Christchurch, New Zealand; ^6^ Centre for Brain Research, The University of Auckland, Auckland, New Zealand; ^7^ University of Auckland, Auckland, New Zealand; ^8^ University Bordeaux, Bordeaux, France

## Abstract

**Background:**

Most Parkinson's disease (PD) patients experience cognitive impairment. Normal cognitive function is supported by the neuromodulatory mechanisms of the cholinergic system. The primary source of cortical cholinergic input are projections from the nucleus basalis of Meynert (NBM) in the basal forebrain. There is evidence that NBM integrity and PET cortical cholinergic function are associated with cognition in PD. We previously found that the integrity of cortical cholinergic projections, measured using anatomically constrained tractography from diffusion‐weighted MRI (DWI), was associated with cognitive function in PD. Here, we test whether cholinergic projection integrity is associated with longitudinal cognitive function.

**Method:**

We used Bayesian linear mixed effects models to examine the association between longitudinal change in cognition and baseline structural integrity of cholinergic cortical pathways in PD participants (baseline *n* = 101; follow‐up *n* = 59; average follow‐up time = 3.2[0.5] years). Neuropsychological testing examined four cognitive domains. Cortical cholinergic integrity was measured using a data‐driven composite score derived from principal component analysis of three DWI measures of cortical cholinergic projection microstructural integrity from our previous work (mean diffusivity, free water fraction, and fibre density and cross‐section). Models accounted for age and allowed for a varying intercept per participant and varying slope for time.

**Result:**

Baseline cortical cholinergic projection integrity (*β* = 0.19 [0.04,0.34], *p* = 98%) was independently associated with cognition; however, there was no evidence cognitive performance was associated with time in this sample (*β* = 0.13 [‐0.08,0.25], *p* <95%). As such, we did not find evidence of an interaction between baseline cortical cholinergic projection integrity and time (*β* = 0.04 [‐0.09,0.11], *p* <95%)).

**Conclusion:**

We found strong evidence for a modest association between baseline cortical cholinergic integrity and cognition in PD. This suggests that the composite measure of cortical cholinergic projection integrity used in this analysis was consistently related to cognitive performance across assessment timepoints but there was no evidence baseline integrity predicted differential rates of cognitive decline. Combining measures from additional components of the brain cholinergic system, or from other neurotransmitter systems, may provide greater insight into cognitive trajectories in PD.

